# Change in Nutritional Status during Hospitalization and Prognosis in Patients with Heart Failure with Preserved Ejection Fraction

**DOI:** 10.3390/nu14204345

**Published:** 2022-10-17

**Authors:** Akihiro Sunaga, Shungo Hikoso, Takahisa Yamada, Yoshio Yasumura, Shunsuke Tamaki, Masamichi Yano, Takaharu Hayashi, Yusuke Nakagawa, Akito Nakagawa, Masahiro Seo, Hiroyuki Kurakami, Tomomi Yamada, Tetsuhisa Kitamura, Taiki Sato, Bolrathanak Oeun, Hirota Kida, Yohei Sotomi, Tomoharu Dohi, Katsuki Okada, Hiroya Mizuno, Daisaku Nakatani, Yasushi Sakata, on behalf of the OCVC-Heart Failure Investigators

**Affiliations:** 1 Department of Cardiovascular Medicine, Osaka University Graduate School of Medicine, 2-2 Yamadaoka, Suita 565-0871, Japan; 2 Division of Cardiology, Osaka General Medical Center, 3-1-56 Mandaihigashi, Sumiyoshi-ku, Osaka 558-8558, Japan; 3 Division of Cardiology, Amagasaki Chuo Hospital, 1-12-1 Shioe, Amagasaki 661-0976, Japan; 4 Department of Cardiology, Rinku General Medical Center, 2-23 Ourai-kita, Rinku, Izumisano 598-8577, Japan; 5 Division of Cardiology, Osaka Rosai Hospital, 1179-3 Nagasonecho, Kitaku, Sakai 591-8025, Japan; 6 Cardiovascular Division, Osaka Police Hospital, 10-31 Kitayamacho, Tennojiku, Osaka 543-0035, Japan; 7 Division of Cardiology, Kawanishi City Hospital, 5-21-1, Kawanishi 666-0195, Japan; 8 Department of Medical Informatics, Osaka University Graduate School of Medicine, 2-2 Yamadaoka, Suita 565-0871, Japan; 9 Department of Medical Innovation, Osaka University Hospital, 2-15 Yamadaoka, Suita 565-0871, Japan; 10 Department of Social and Environmental Medicine, Osaka University Graduate School of Medicine, 2-2 Yamadaoka, Suita 565-0871, Japan

**Keywords:** heart failure with preserved ejection fraction, nutritional status, GNRI, malnutrition

## Abstract

The impact of changes in nutritional status during hospitalization on prognosis in patients with heart failure with preserved ejection fraction (HFpEF) remains unknown. We examined the association between changes in the Geriatric Nutritional Risk Index (GNRI) and prognosis during hospitalization in patients with HFpEF stratified by nutritional status on admission. Nutritional status did and did not worsen in 348 and 349 of 697 patients with high GNRI on admission, and in 142 and 143 of 285 patients with low GNRI on admission, respectively. Kaplan–Meier analysis revealed no difference in risk of the composite endpoint, all-cause death, or heart failure admission between patients with high GNRI on admission whose nutritional status did and did not worsen. In contrast, patients with low GNRI on admission whose nutritional status did not worsen had a significantly lower risk of the composite endpoint and all-cause death than those who did. Multivariable analysis revealed that worsening nutritional status was independently associated with a higher risk of the composite endpoint and all-cause mortality in patients with low GNRI on admission. Changes in nutritional status during hospitalization were thus associated with prognosis in patients with malnutrition on admission, but not in patients without malnutrition among those with HFpEF.

## 1. Introduction

Malnutrition is common in patients with heart failure and is associated with poor prognosis [1,2], making assessment and management of nutritional status important for the treatment of heart failure. A number of nutritional assessment tools have been developed [3,4,5,6]. Among them, the Geriatric Nutritional Risk Index (GNRI), which is calculated based on albumin and body mass index [4], is commonly used to assess nutritional status in patients with heart failure [7,8,9]. GNRI is supposed to be the most appropriate nutritional assessment tool for patients with heart failure, since GNRI is thought to be less affected by a change of volume which accompanies heart failure treatment [10] and has the best prognostic value among several nutritional assessment tools [2].

Pathophysiology of heart failure with preserved ejection fraction (HFpEF) is heterogeneous. Malnutrition has been thought to be one of the causes of HFpEF [11] as well as a prognostic factor. Nutritional status at admission or discharge assessed by GNRI is reportedly associated with prognosis in patients with HFpEF [10,12]. Hospitalized elderly patients are at high risk of malnutrition and are more likely to be worse off after admission than when they were admitted [13]. Elderly patients are more likely to have HFpEF [14]. However, the impact of changes in nutritional status during hospitalization on prognosis in patients with HFpEF remains unknown. While it is important to examine the relationship between changes in nutritional status and prognosis to assess whether interventions in nutritional status can improve prognosis, it should be noted that baseline nutritional status itself has a strong prognostic impact. Therefore, the effect of changes in nutritional status may differ according to patients’ nutritional status at admission.

The purpose of this study is to determine the association between changes in GNRI and prognosis during hospitalization in patients with HFpEF stratified by nutritional status on admission.

## 2. Materials and Methods

### 2.1. Study Patients

Of the 1095 patients registered in the prospective, multicenter, observational study of patients with HFpEF (PURSUIT-HFpEF) registry [15] between June 2016 and January 2021, 86 patients without GNRI, 12 patients with in-hospital death, and 15 patients with amyloidosis, chronic thromboembolic pulmonary hypertension or pulmonary arterial hypertension were excluded. A total of 982 patients were studied. The registry, which started in June 2016, enrolled patients hospitalized with a diagnosis of decompensated heart failure based on the Framingham criteria and who met the following criteria: left ventricular ejection fraction (LVEF) ≥ 50% on a transthoracic cardiac echocardiographic (TTE) test on admission and *N*-terminal pro-brain natriuretic peptide (NT-proBNP) ≥ 400 pg/mL or brain natriuretic peptide ≥ 100 pg/mL on admission. We excluded patients with severe aortic stenosis, aortic regurgitation, mitral stenosis, or mitral regurgitation due to structural changes in the valve detected by TTE on admission. We also excluded patients under 20 years old, patients with acute coronary syndrome on admission, patients with poor life prognoses within six months due to non-cardiac diseases, patients who had received a heart transplant, and patients considered not to be appropriate for the study by the attending physician. Thirty-one facilities participated in this study. We did not have any protocol for nutritional treatment after discharge.

All patients provided written informed consent for participation in this study, which was approved by the ethics committee of each participating hospital. This study followed the ethical guidelines outlined by the Helsinki Declaration. The study protocol was approved by the Institutional Review Board of all participating facilities.

### 2.2. Data Collection

We collected data such as detailed past history, accompanying diseases, quality of life, Clinical Frailty Scale [16], medication history, and laboratory and echocardiographic data. Each patient was followed to collect outcome data such as mortality, cause of death, number of hospitalizations, and cause of hospitalization.

Change in sodium level was calculated as sodium at discharge—sodium on admission. Change in hemoglobin level was calculated as hemoglobin level as discharge—hemoglobin level on admission.

In echocardiography, inferior vena cava diameter was measured using a standard method. LVEF was measured using the Simpson method. Left ventricular mass index (LVMI) was calculated using the left ventricular diastolic diameter, left ventricular posterior thickness, interventricular septum thickness, and body surface area. E/e’ was the mean of septal E/e’ and lateral E/e’. The tricuspid pressure gradient was determined using the simplified Bernoulli equation.

Plasma volume was calculated using Hakim formula as follows: (1 − hematocrit) × [a + (b × body weight)] (a = 1530 in males and a = 864 in females, b = 41.0 in males and b = 47.9 in females) [17]. Change in plasma volume was calculated by plasma volume at discharge–plasma volume on admission.

Prognostic nutrition index (PNI) [1] and controlling nutritional status (CONUT) [5] score were calculated on admission and at discharge. Change in PNI was calculated as PNI at discharge–PNI on admission. Change in CONUT score was calculated as CONUT score at discharge–CONUT score on admission.

Research cardiologists and specialized research nurses recorded the patients’ data during their hospital stay. In-hospital data were transferred to the data collection center for processing and analysis. Medical history was obtained on admission. Vital signs, body mass index (BMI), echocardiography, laboratory data, and medication use were obtained both on admission and at discharge.

### 2.3. GNRI

**Figure 1:**
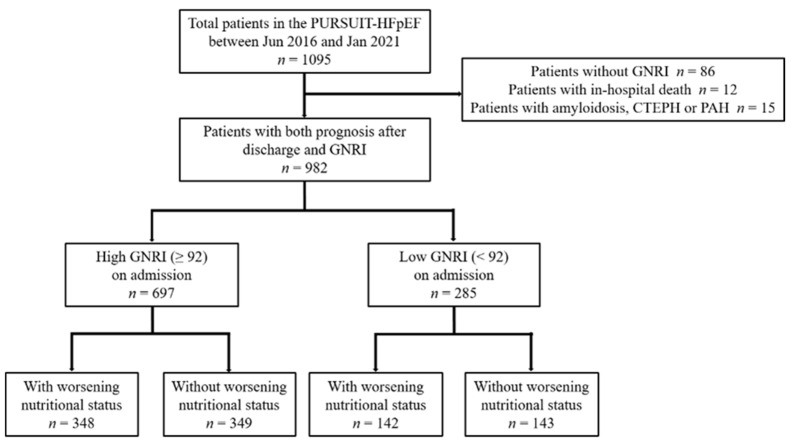
Patient selection. GNRI, geriatric nutritional risk index; CTEPH, chronic thromboembolic pulmonary hypertension; PAH, pulmonary arterial hypertension.

GNRI was calculated as follows: 14.89 × albumin (g/dL) + 41.7 × BMI (kg/m^2^)/22. GNRI was calculated both on admission and at discharge. Delta GNRI was calculated as GNRI at discharge–GNRI on admission. GNRI was classified based on the risk of malnutrition as none (>98), mild (92 to 98), moderate (82 to <92), or severe (<82) [4]. First, we divided the participants into two groups based on whether their GNRI was high (≥92) or low (<92). Each group was then further dichotomized into high and low according to the median delta GNRI (Figure 1).

### 2.4. Statistical Analysis

Continuous variables are expressed as median (interquartile range). Categorical data are presented as percentages unless otherwise specified. Tests for significance were conducted using the unpaired *t*-test, Mann–Whitney U test or Wilcoxon signed-rank test for continuous variables, and the chi-squared test or Fisher’s exact test for categorical variables. The primary endpoint of this study was the composite of all-cause mortality and heart failure admission for 2 years. Secondary endpoints were all-cause mortality and heart failure admission for 2 years. Endpoints were estimated using Kaplan–Meier curves and statistical differences were determined using the log-rank test. Univariable analysis and multivariable analysis using a Cox proportional hazards regression model were also performed. The multivariable analysis was adjusted for age, sex, history of heart failure hospitalization, hypertension, diabetes, hemoglobin, estimated glomerular filtration rate, *N*-terminal pro-brain natriuretic peptide level, and use of angiotensin-converting enzyme inhibitor or angiotensin Ⅱ receptor blocker. These covariates are well-established predictors of risk in patients with HFpEF [18,19]. Adjusted hazard ratios (HRs) and 95% confidence intervals (CIs) were calculated for each endpoint using Cox proportional hazards regression models. All statistical analyses were performed using SPSS version 25 (IBM Corp., Armonk, NY, USA). Statistical significance was defined as *p* < 0.05.

## 3. Results

### 3.1. Baseline Characteristics

The median follow-up period was 421 [260, 730] days. Of the 982 patients, 697 had high and 285 had low GNRI on admission. The baseline characteristics and prognosis of these groups are shown in Supplementary Table S1 and Figure S1. As mentioned in the Introduction section, we hypothesized that those whose nutritional status worsened during hospitalization may have a worse prognosis than would be expected based on their nutritional status at admission, whereas those whose nutritional status did not worsen may have a better prognosis.

**Figure 2:**
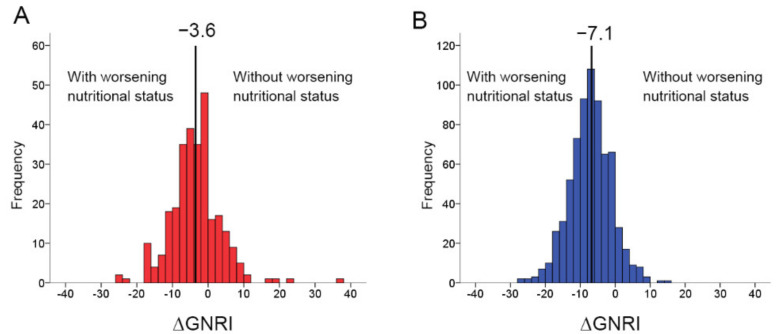
Distribution of delta GNRI stratified by low or high GNRI on admission. The median delta GNRI value in patients with low GNRI on admission was −3.6 (**A**) and that in patients with high GNRI on admission was −7.1 (**B**). GNRI, geriatric nutritional risk index.

To determine the association between changes in GNRI during hospitalization and prognosis in patients with HFpEF stratified by nutritional status on admission, we divided the patients into two groups: those with high GNRI on admission and those with low GNRI on admission. The distribution of the patients’ delta GNRI stratified by high or low GNRI on admission is shown in Figure 2. The median delta GNRI in patients with high GNRI on admission was −7.1 and that in patients with low GNRI on admission was −3.6. We further divided the 697 patients with high GNRI on admission into those whose nutritional status worsened (delta GNRI < −7.1) and those that did not (delta GNRI ≥ −7.1), and 285 patients with low GNRI on admission into the same categories (delta GNRI < −3.6 vs. delta GNRI ≥ −3.6).

**Figure 3:**
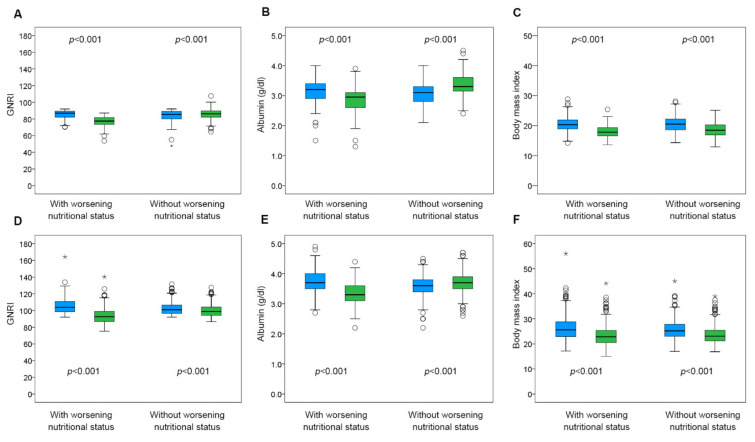
Change in GNRI, albumin and BMI during hospitalization. The blue bar shows the value on admission and the green bar shows that at discharge. (**A**–**C**) show the values of patients with low GNRI on admission, and (**D**–**F**) show the values of patients with high GNRI on admission. Asterisk indicates the value under 1st quartile − 3 × inter quartile range and the value over 3rd quartile + 3 × inter quartile range.

The baseline characteristics of these four groups are shown in Table 1. Patients with high GNRI and low GNRI on admission whose nutritional status worsened had significantly lower hemoglobin and albumin. Among patients with high GNRI on admission, patients with worsening nutritional status showed older age, higher frequency of NYHA ≥II and use of calcium channel blocker, higher LVMI, sodium level and NT-proBNP level, and lower hemoglobin and albumin level. Among patients with low GNRI on admission, patients with worsening nutritional status showed older age, lower BMI, lower frequency of diabetes mellitus, use of aldosterone antagonist, higher sodium level, and lower hemoglobin and albumin levels. The comparison of GNRI, albumin, and BMI on admission and at discharge among the four groups is shown in Figure 3. While albumin levels at discharge were higher than that on admission in patients with high and low GNRI on admission whose nutritional status did not worsen, they were lower in both patients with high and low GNRI on admission whose nutritional status worsened. BMI at discharge was lower than that on admission in all groups.

Change in patients’ condition during admission was shown in Supplementary Table S2. The change in plasma volume between admission and discharge was not significantly different between patients with and without worsening nutritional status among both patients with high and low GNRI on admission. In patients with high GNRI on admission, there was no significant difference in the change in serum sodium between patients with worsening nutritional status and those without. In patients with low GNRI on admission, patients with worsening nutritional status had a greater decrease in serum sodium than those without. Patients with worsening nutritional status had a greater decrease in hemoglobin levels than those without among both patients with high and low GNRI on admission. Patients with worsening nutritional status had a greater decrease in PNI and a greater increase in CONUT score than those without worsening nutritional status among both patients with high and low GNRI on admission. (Supplementary Table S2).

### 3.2. Outcomes

**Figure 4:**
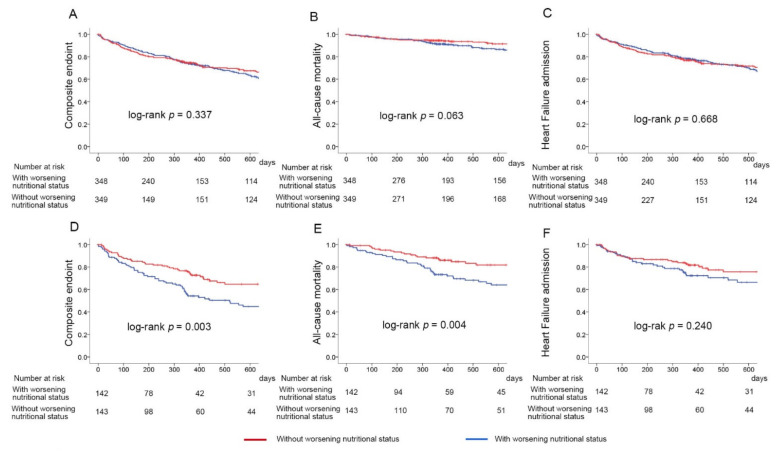
Comparison of outcomes between patients whose nutritional status did and did not worsen stratified by high or low GNRI on admission. (**A**–**C**) show the survival curves of patients with high GNRI on admission, and (**D**–**F**) show the survival curves of patients with low GNRI on admission. GNRI, geriatric nutritional risk index.

Kaplan–Meier analysis showed that patients with low GNRI on admission had a higher risk of the composite endpoint and all-cause mortality, but a similar risk of heart failure hospitalization to those with high GNRI on admission (Supplementary Figure S1). In patients with high GNRI on admission, Kaplan–Meier analysis at follow-up of 2 years revealed no significant difference in risk of the composite endpoint, all-cause death, or heart failure admission between patients whose nutritional status did and did not worsen. In patients with low GNRI on admission, Kaplan–Meier analysis at follow-up of 2 years revealed that those whose nutritional status worsened had a significantly higher risk of the composite endpoint and all-cause death than those who did not (Figure 4).

In patients with high GNRI on admission, Kaplan–Meier analysis until 6 months after discharge revealed no significant difference in risk of the composite endpoint (log-rank *p* = 0.425), all-cause death (log-rank *p* = 0.995), or heart failure admission (log-rank *p* = 0.400) between patients whose nutritional status did and did not worsen. In patients with low GNRI on admission, Kaplan–Meier analysis at follow-up of 6 months revealed that those whose nutritional status worsened tended to have a higher risk of the composite endpoint (log-rank *p* = 0.056) (Supplementary Figure S2).

The incidence rates of all-cause death, cardiac death and non-cardiac death, and heart failure admission in the four groups are shown in Table 2. All-cause death, cardiac death and non-cardiac death, and heart failure admission occurred more frequently in patients with low GNRI on admission and whose nutritional status worsened compared to the other groups but were comparable to those with high GNRI on admission.

Results of the multivariable analysis with a Cox proportional hazard model of the composite endpoint, all-cause mortality, and heart failure admission are shown in Table 3. Worse nutritional status was independently associated with a higher risk of the composite endpoint and all-cause mortality in patients with low GNRI on admission, but not in patients with high GNRI on admission.

## 4. Discussion

### 4.1. Main Findings

We showed that severe worsening nutritional status during treatment for decompensated heart failure was significantly associated with worse prognosis in patients with low GNRI on admission, but not in patients with high GNRI on admission among those with HFpEF. This study is the first to report the impact of changes in nutritional status on prognosis in patients with HFpEF. Our findings may suggest the importance of avoiding worsening nutritional status for preventing poor prognosis, especially in patients with malnutrition.

### 4.2. Previous Studies

We clarified that the progression of malnutrition has prognostic impacts in patients with malnutrition at baseline in patients with HFpEF. A number of previous studies have reported the role of malnutrition in patients with heart failure including HFpEF. Minamisawa et al. reported that patients with HFpEF are at an elevated risk for malnutrition, which was associated with an increased risk for CV events in 1677 patients enrolled in the American regions of the TOPCAT trial [9]. Chien et al. examined 1120 patients and reported that malnutrition was frequently and strongly associated with systemic inflammation in Asian patients hospitalized for acute HFpEF [20]. Hirose et al. examined 201 patients with HFrEF and 250 patients with HFpEF and reported that among patients with acute decompensated HF, assessment of nutritional status with GNRI is useful for stratifying patients at high risk for longer length of hospital stay in HFpEF but not in HFrEF [21]. Watanabe et al. analyzed 420 patients and reported that inflammation was associated with malnutrition in HFmrEF and HFpEF, while congestion was an independent predictor of malnutrition in HFrEF [22]. Nishi et al. examined 110 patients and reported that nutritional screening using the GNRI at discharge is helpful to predict the long-term prognosis of elderly HFpEF patients [12]. All these previous reports examined the association between malnutrition and heart failure including HFpEF at a one-time point. However, there have been no reports about the association between change in nutrition status during hospitalization and prognosis in patients with HFpEF.

### 4.3. Cardiac Cachexia and Malnutrition

In this study, nearly 30% of patients showed malnutrition on admission, and a considerable number of patients showed worsening nutrition during hospitalization. In heart failure patients, cardiac cachexia is common. Increases in catecholamines, inflammatory cytokines, and insulin resistance result in protein catabolism, lipolysis, and bone loss [23,24]. Cardiac cachexia and malnutrition are closely related. In particular, acute heart failure increases the risk of malnutrition due to decreased albumin production due to hepatic congestion, decreased absorption of nutrients due to intestinal edema, and decreased food intake. All of these factors lead to hypermetabolism, impaired feeding and absorption, and finally malnutrition [25,26,27]. Heart failure leads to malnutrition, and malnutrition further exacerbates heart failure. Since patients with heart failure are likely to be malnourished [1,2], their nutritional status should be monitored during heart failure treatment.

### 4.4. Assessment of Nutritional Status during Hospitalization

We used the GNRI to assess nutritional status in this study. While several other tools such as the Malnutrition Universal Screening Tool, Subjective Global Assessment, and Nutritional Risk Screening 2002 [28] have also been used to assess nutritional status, the results of these measures may be influenced by the experience of the examiner because they involve subjective assessments. Further, CONUT [5] and PNI [1], two simple and objective measures that include lymphocyte counts may be unsuitable for examining nutritional status in the acute phase, since the infection was frequently accompanied in patients with acute decompensated heart failure.

In contrast, GNRI may be the most appropriate for assessing changes in nutritional status during hospitalization, because it is less affected by heart failure treatment. Since volume reduction results in a decrease in BMI and an increase in albumin, the effects of volume reduction may be minimized in changes in GNRI, and we expect that changes in GNRI mainly reflect nutritional status in our study. Actually, our data indicated that a decrease in plasma volume has tended to be less in patients with worsening nutritional status than in those without nutritional status in both groups (Supplementary Table S2), suggesting that change in GNRI does not correlate with the change in volume. Moreover, change in hemoglobin level, PNI, and CONUT score had decreased more in patients with worsening nutritional status than those without nutritional status in both groups (Supplementary Table S2). All these findings suggest that the change in GNRI in this study reflected the true occurrence of new malnutrition rather than a measurement bias due to volume reduction with heart failure treatment, whereas we cannot rule out the possibility that volume reduction may affect these changes to some extent.

### 4.5. Relationship between Change in Nutritional Status and Prognosis

Our data suggest that a worsening nutritional status during hospitalization had a negative impact on long-term prognosis in patients with low GNRI on admission, but not in those with high GNRI on admission. Since malnutrition on admission is reportedly associated with poor prognosis in patients with heart failure [10], assessment of nutritional status on admission is important for risk stratification and should be routinely performed. The usefulness of risk assessment based on nutrition has also been reported in outpatients [9]. Our data further emphasize the importance of continuous monitoring and effort to avoid worsening nutritional status during treatment for decompensated heart failure. On the other hand, the association between the change in nutritional status and the shorter-term prognosis was less significant. This may be due to a lack of statistical power resulting from the smaller number of events. Another possibility is that a change of GNRI during hospitalization may have more impact on the long-term prognosis than the short-term prognosis.

It is interesting that the impact of a deterioration in nutritional status differed according to baseline nutritional status. This may be due to a difference in reserve capacity for nutritional conditions between patients with and without malnutrition. Malnutrition can cause hypoproteinemia and weakened immunity, leading to exacerbation of heart failure and infections [29]. Conversely, worsening heart failure causes a deterioration of patients’ nutritional status due to intestinal edema and reduced food intake, creating a vicious cycle [24,25,30]. Patients with malnutrition on admission can more easily enter this vicious cycle than those without malnutrition, which may explain the poor prognosis in only malnourished patients. Careful monitoring and management of nutritional status are especially important in these populations.

Our data may also imply that maintaining or improving nutritional status can improve prognosis in patients with malnutrition on admission. Nutritional support has been reported to improve nutritional status in a meta-analysis [31]. Nutritional therapy was associated with lower mortality rates than conventional treatment in a population in which about 25% of patients had heart failure [32]. Nutritional interventions have been reported to improve LVEF and decrease NT-proBNP by adjusting inflammatory levels [33]. Our current study is in line with these previous reports, and these findings suggest that nutritional interventions during hospitalization that do not worsen nutritional status may improve prognosis in patients with HFpEF. Further prospective studies are needed to examine the effect of nutritional intervention on prognosis. Regarding nutritional strategies to intervene against malnutrition, the use of individualized nutritional support to reach protein and caloric goals during the hospital stay improved important clinical outcomes, including survival, compared with standard hospital food in medical inpatients at nutritional risk [34]. A previous study reported that nutritional support alone may be insufficient as an intervention and that nutritional support in combination with exercise therapy may be more effective [35]. Appropriate interventions for these populations should be also investigated.

### 4.6. Limitations

We only used the GNRI to assess nutritional status. Additional studies using other nutritional indicators might be useful to confirm our findings. Further studies are also needed to determine whether nutritional interventions improve prognosis.

## 5. Conclusions

Worsening nutritional status during hospitalization was associated with prognosis in patients with low GNRI on admission, but not in patients with high GNRI on admission among those with HFpEF. Nutritional assessment by GNRI on admission to identify patients with malnutrition, and interventions on nutritional status in these populations may be useful for improving prognosis in patients with HFpEF.

## Figures and Tables

**Table 1:** Baseline characteristics.

	High GNRI on Admission			Low GNRI on Admission		
Variable	With Worsening Nutritional Status *n* = 348	Without Worsening Nutritional Status *n* = 349	*p*	With Worsening Nutritional Status *n* = 142	Without Worsening Nutritional Status *n* = 143	*p*
Age, years	83 (78, 87)	81 (75, 86)	0.001	85 (78, 90)	83 (78, 88)	0.043
Male, *n* (%)	162 (46.6)	174 (49.9)	0.383	49 (34.5)	62 (43.4)	0.126
Body mass index, kg/m^2^	22.8 (20.5, 25.4)	23.1 (21.2, 25.4)	0.214	17.8 (16.6, 19.3)	18.5 (16.9, 20.3)	0.016
Current smoking, *n* (%)	31 (9.1)	39 (11.2)	0.625	12 (8.6)	19 (13.4)	0.323
NYHA ≥ 2, *n* (%)	222 (64.3)	187 (54.4)	0.008	103 (74.1)	103 (73.6)	0.920
Systolic blood pressure, mmHg	120 (106, 132)	118 (108, 130)	0.715	118 (105, 134)	118 (103, 129)	0.623
Heart rate, bpm	69 (61, 78)	69 (60, 77)	0.801	74 (64, 81)	72 (64, 80)	0.554
Prior heart failure admission, %	79 (23.4)	86 (25.0)	0.635	29 (20.9)	35 (24.6)	0.449
Hypertension, *n* (%)	314 (90.2)	302 (86.5)	0.128	108 (76.6)	110 (76.9)	0.948
Diabetes mellitus, *n* (%)	126 (36.4)	134 (38.5)	0.570	24 (17.1)	39 (27.7)	0.035
Dyslipidemia, *n* (%)	156 (45.1)	170 (49.0)	0.303	46 (32.6)	40 (28.4)	0.438
Stroke, *n* (%)	55 (15.9)	51 (14.7)	0.673	15 (10.6)	21 (14.8)	0.295
Atrial fibrillation, *n* (%)	165 (47.4)	172 (49.3)	0.621	57 (40.1)	55 (38.5)	0.772
Chronic kidney disease, *n* (%)	146 (42.2)	141 (40.6)	0.676	51 (35.9)	51 (36.2)	0.964
Malignant disease, *n* (%)	36 (10.4)	47 (13.7)	0.193	16 (11.5)	23 (16.2)	0.256
LVEF, %	61 (55, 65)	61 (56, 65)	0.761	61 (56, 66)	60 (56, 66)	0.655
Left atrial diameter, mm	45 (40, 50)	46 (40, 51)	0.128	41 (36, 46)	40 (35, 46)	0.485
LVMI, g/m^2^	108 (89, 129)	102 (85, 125)	0.043	100 (83, 119)	95 (77, 114)	0.l18
Mean E/e’	13 (10, 17)	12 (10, 17)	0.641	13 (10, 16)	11 (9, 15)	0.082
TRPG, mmHg	27 (22, 32)	27 (22, 33)	0.768	26 (22, 32)	27 (22, 33)	0.153
IVC diameter, mm	14.0 (11.4, 17.1)	14.0 (11.0, 17.2)	0.942	13.2 (10.0, 16.7)	12.4 (9.8, 15.0)	0.108
Sodium, mEq/L	140 (137, 142)	139 (137, 141)	0.024	140 (137, 141)	139 (135, 141)	0.045
Hemoglobin, g/dL	11.1 (9.7, 12.5)	12.0 (10.8, 13.5)	<0.001	10.5 (9.2, 11.7)	11.1 (10.1, 12.3)	<0.001
Creatinine, mg/dL	1.1 (0.9, 1.6)	1.1 (0.9, 1.5)	0.754	1.0 (0.8, 1.4)	1.1 (0.8, 1.5)	0.322
eGFR, mL/min/1.73 m^2^	40.3 (29.0, 54.4)	41.5 (31.6, 53.8)	0.428	42.6 (32.6, 58.4)	44.8 (29.2, 57.0)	0.901
Albumin, g/dL	3.3 (3.1, 3.6)	3.7 (3.5, 3.9)	<0.001	3.0 (2.6, 3.1)	3.3 (3.1, 3.6)	<0.001
NT-proBNP, pg/mL	1100 (586, 2375)	803 (373, 1797)	<0.001	1350 (562, 3080)	1399 (506, 2607)	0.498
ACE-I or ARB, *n* (%)	211 (60.6)	193 (55.3)	0.154	58 (40.8)	73 (51.0)	0.084
Calcium channel blocker, *n* (%)	192 (55.3)	162 (46.4)	0.019	61 (43.0)	61 (42.7)	0.959
Beta blocker, *n* (%)	199 (57.3)	194 (55.6)	0.639	75 (52.8)	81 (56.6)	0.516
Diuretics, *n* (%)	283 (81.3)	294 (84.2)	0.307	110 (77.5)	118 (82.5)	0.286
Aldosterone antagonist, *n* (%)	130 (37.4)	143 (41.0)	0.328	48 (33.8)	66 (46.2)	0.033
Statin, *n* (%)	122 (35.2)	132 (37.8)	0.465	34 (23.9)	46 (32.2)	0.122
Hospital stay, days	17 (13, 23)	15 (12, 19)	0.003	19 (13, 27)	17 (12, 26)	0.313
Quality of life score	0.776 (0.587, 0.895)	0.825 (0.667, 1.000)	0.001	0.709 (0.491, 0.869)	0.732 (0.504, 0.875)	0.893
Clinical Frailty Scale	3 (3, 5)	3 (2, 4)	0.028	4 (3, 6)	4 (3, 6)	0.046

GNRI, geriatric nutritional risk index; LVEF, left ventricular ejection fraction; LVMI, left ventricular mass index; TRPG, tricuspid regurgitation pressure gradient; IVC, inferior vena cava; eGFR, estimated glomerular filtration rate; NT-proBNP, *N*-terminal pro-brain natriuretic peptide; ACE-I, angiotensin converting enzyme inhibitor; ARB, angiotensin Ⅱ receptor blocker.

**Table 2:** Incident rate of endpoint.

	High GNRI on Admission						Low GNRI on Admission					
	With Worsening Nutritional STATUS			Without Worsening Nutritional Status			With Worsening Nutritional Status			Without Worsening Nutritional Status		
	Number of Events	Person-Years	IR	Number of Events	Person-Years	IR	Number of Events	Person-Years	IR	Number of Events	Person-Years	IR
Composite endpoint	104	365.5	28.6	91	366.6	24.8	60	116.6	51.1	41	147.4	28.1
All-cause death	38	435.7	8.8	24	445.8	5.5	40	143.9	27.8	21	165.8	12.9
HF admission	84	365.5	22.8	80	366.6	21.9	30	116.6	25.6	27	147.4	18.4

GNRI, geriatric nutritional risk index; IR, incident rate; HF, heart failure.

**Table 3:** Hazard ratio of no worsening nutritional status for each endpoint.

	Unadjusted HR (95% CI)	*p*	Adjusted HR (95% CI)	*p*
High GNRI on admission				
Composite endpoint	1.15 (0.87–1.52)	0.337	1.01 (0.73–1.39)	0.974
All–cause death	1.60 (0.97–2.65)	0.065	1.69 (0.92–3.04)	0.094
Heart failure admission	1.07 (0.79–1.45)	0.668	0.92 (0.65–1.30)	0.629
Low GNRI on admission				
Composite endpoint	1.79 (1.21–2.65)	0.004	1.65 (1.05–2.59)	0.030
All–cause death	2.10 (1.25–3.54)	0.004	1.84 (1.04–3.26)	0.038
Heart failure admission	1.36 (0.81–2.30)	0.243	1.37 (0.75–2.49)	0.309

Adjusted for age, sex, history of heart failure hospitalization, hypertension, diabetes, hemoglobin, eGFR, NT-proBNP, ACE-I/ARB, HR, hazard ratio; CI, confidence interval; GNRI, geriatric nutritional risk index, eGFR, estimated glomerular filtration rate; NT-proBNP, *N*-terminal pro-brain natriuretic peptide; ACE-I, angiotensin-converting enzyme inhibitor; ARB, angiotensin II receptor blocker.

## Data Availability

Not applicable.

## Supplementary Materials

The following supporting information can be downloaded at: https://www.mdpi.com/article/10.3390/nu14204345/s1, Figure S1: Comparison of outcomes between patients with high or low GNRI on admission.; Figure S2: Comparison of outcomes until 6 months after discharge between patients whose nutritional status did and did not worsen stratified by high or low GNRI on admission; Table S1: Baseline characteristics grouped by GNRI; Table S2: Change in patients’ condition during admission.
