# Supplementary material for: Change in Nutritional Status during Hospitalization and Prognosis in Patients with Heart Failure with Preserved Ejection Fraction
Source: Nutrients. 2022 Oct 17;14(20):4345. doi: 10.3390/nu14204345 (PMC9611174; doi:10.3390/nu14204345)
Supplement: Supplementary file 1 [file nutrients-14-04345-s001.zip › Supple_Table S1_20220817.pdf]

Supplementary Table S1. Baseline characteristics grouped by GNRI

Variable	High GNRI on admission n=697	Low GNRI on admission n=285	P
Age, years	82 [77, 86]	84 [78, 89]	<0.001
Male, %	48	39	0.008
Body mass index, kg/m^2	23.0 [20.9, 25.4]	18.1 [16.7, 19.8]	<0.001
Current smoking, %	10	11	0.042
Systolic blood pressure, mmHg	119 [107, 131]	118 [105, 132]	0.331
Heart rate, bpm	69 [61, 77]	72 [64, 81]	<0.001
Prior heart failure admission, %	24	23	0.630
Hypertension, %	88	77	<0.001
Diabetes mellitus, %	38	22	<0.001
Dyslipidemia, %	47	31	<0.001
Stroke, %	15	13	0.297
Atrial fibrillation, %	48	39	0.010
Chronic kidney disease, %	41	36	0.120
Malignant disease, %	12	14	0.435
LVEF, %	61 [55, 65]	61 [56, 66]	0.691
Left atrial diameter, mm	45 [40, 50]	40 [36, 46]	<0.001
Left ventricular mass index, g/m^2	105 [87, 127]	97 [80, 117]	<0.001
E/e’	12.5 [9.9, 16.9]	11.8 [9.1, 15.6]	0.029
Inferior vena cava diameter, mm	14 [11, 17]	13 [10, 16]	<0.001
Sodium, mEq/L	137 [135, 140]	136 [134, 139]	0.014
Hemoglobin, g/dL	11.6 [10.3, 13.0]	10.8 [9.7, 12.0]	<0.001
Creatinine, mg/dL	1.1 [0.9, 1.5]	1.0 [0.8, 1.5]	0.007
eGFR, ml/min/1.73m^2	41.1 [29.8. 54.0]	44.1 [, 57]	0.103
Albumin, g/dL	3.5 [3.2, 3.8]	3.2 [2.8, 3.5]	<0.001
NT-pro BNP, pg/mL	946 [460, 2155]	1387 [531, 2790]	0.002
ACE-I or ARB, %	58	46	0.001
Calcium channel blocker, %	51	43	0.022
Beta blocker, %	57	55	0.620
Diuretics, %	83	80	0.303
Aldosterone antagonist, %	39	40	0.809
Statin, %	37	28	0.011

GNRI, geriatric nutritional risk index; LVEF, left ventricular ejection fraction; eGFR, estimated glomerular filtration rate; NT-proBNP, N-terminal pro-brain natriuretic peptide; ACE-I, angiotensin converting enzyme inhibitor; ARB, angiotensin Ⅱ receptor blocker.
