# Supplementary material for: Change in Nutritional Status during Hospitalization and Prognosis in Patients with Heart Failure with Preserved Ejection Fraction
Source: Nutrients. 2022 Oct 17;14(20):4345. doi: 10.3390/nu14204345 (PMC9611174; doi:10.3390/nu14204345)
Supplement: Supplementary file 1 [file nutrients-14-04345-s001.zip › Supple_Table S2_20220826.pdf]

Supplementary Table S2. Change in patients' condition during admission

	High GNRI on admission			Low GNRI on admission		
	With worsening nutritional status n = 348	Without worsening nutritional status n = 349	P	With worsening nutritional status n = 142	Without worsening nutritional status n = 143	P
Change in plasma volume	-167 [-317, -8]	-194 [-335, -60]	0.057	-160 [-270, -43]	-198 [-323, -77]	0.070
Change in serum sodium	0 [-3.0, 2.0]	-1.0 [-3.0, 1.0]	0.088	0 [-3.0, 3.0]	-1.0 [-4.0, 2.0]	0.043
Change in hemoglobin level	-0.3 [-1.2, 0.8]	0.6 [-0.2, 1.5]	<0.001	-0.2 [-0.8, 0.6]	0.7 [-0.2, 1.6]	<0.001
Change in PNI	-3.1 [-6.5, -0.7]	2.0 [-0.6, 4.7]	<0.001	-1.0 [-3.9, 1.8]	4.3 [1.5, 6.9]	<0.001
Change in CONUT	1 [-1, 2]	-1 [-2, 0]	<0.001	0 [-1, 2]	-2 [-4, -1]	<0.001

GNRI, geriatric nutritional risk index; PNI, prognostic nutrition index; CONUT, controlling nutritional status.
