# Supplementary figures and images for: Change in Nutritional Status during Hospitalization and Prognosis in Patients with Heart Failure with Preserved Ejection Fraction
Source: Nutrients. 2022 Oct 17;14(20):4345. doi: 10.3390/nu14204345 (PMC9611174; doi:10.3390/nu14204345)

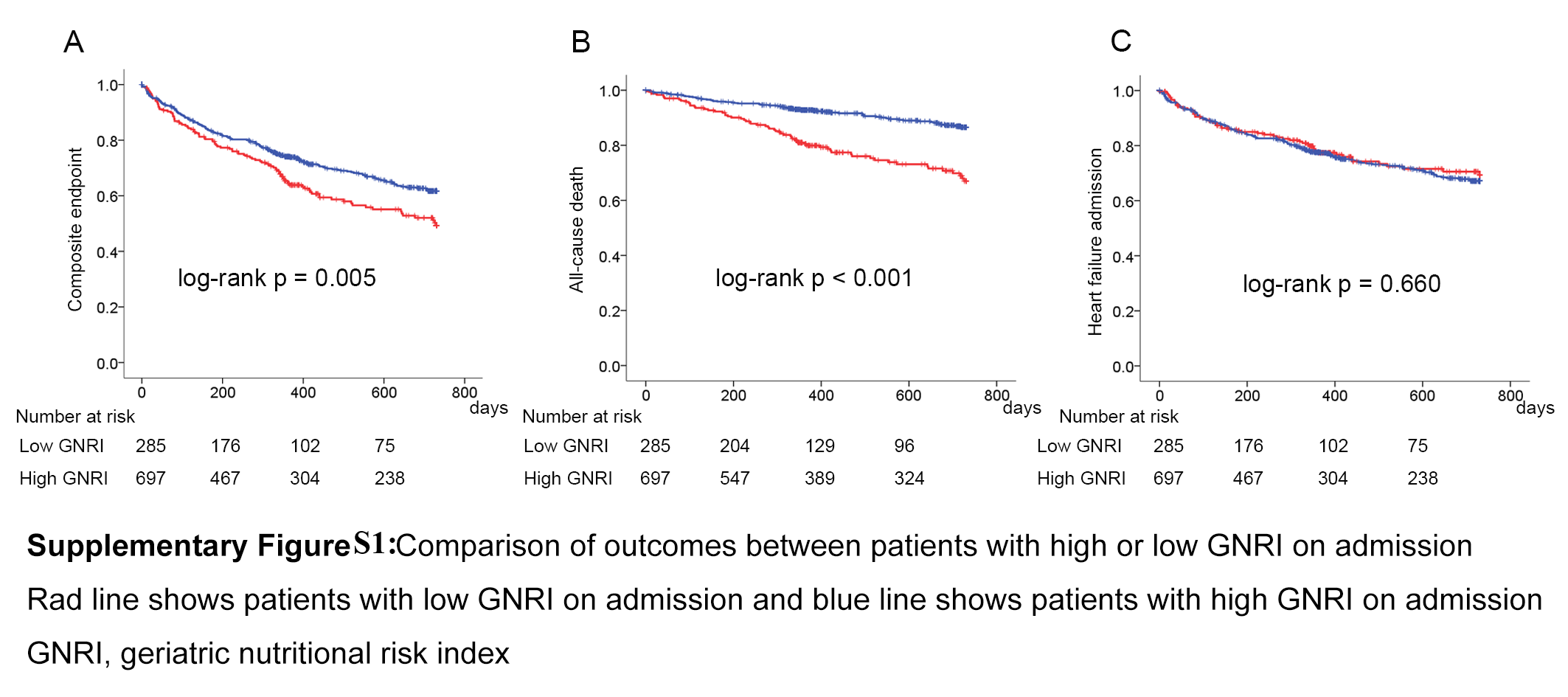

Supplement: Supplementary file 1 [file nutrients-14-04345-s001.zip › Suppl Figure S1_20220826.tif]

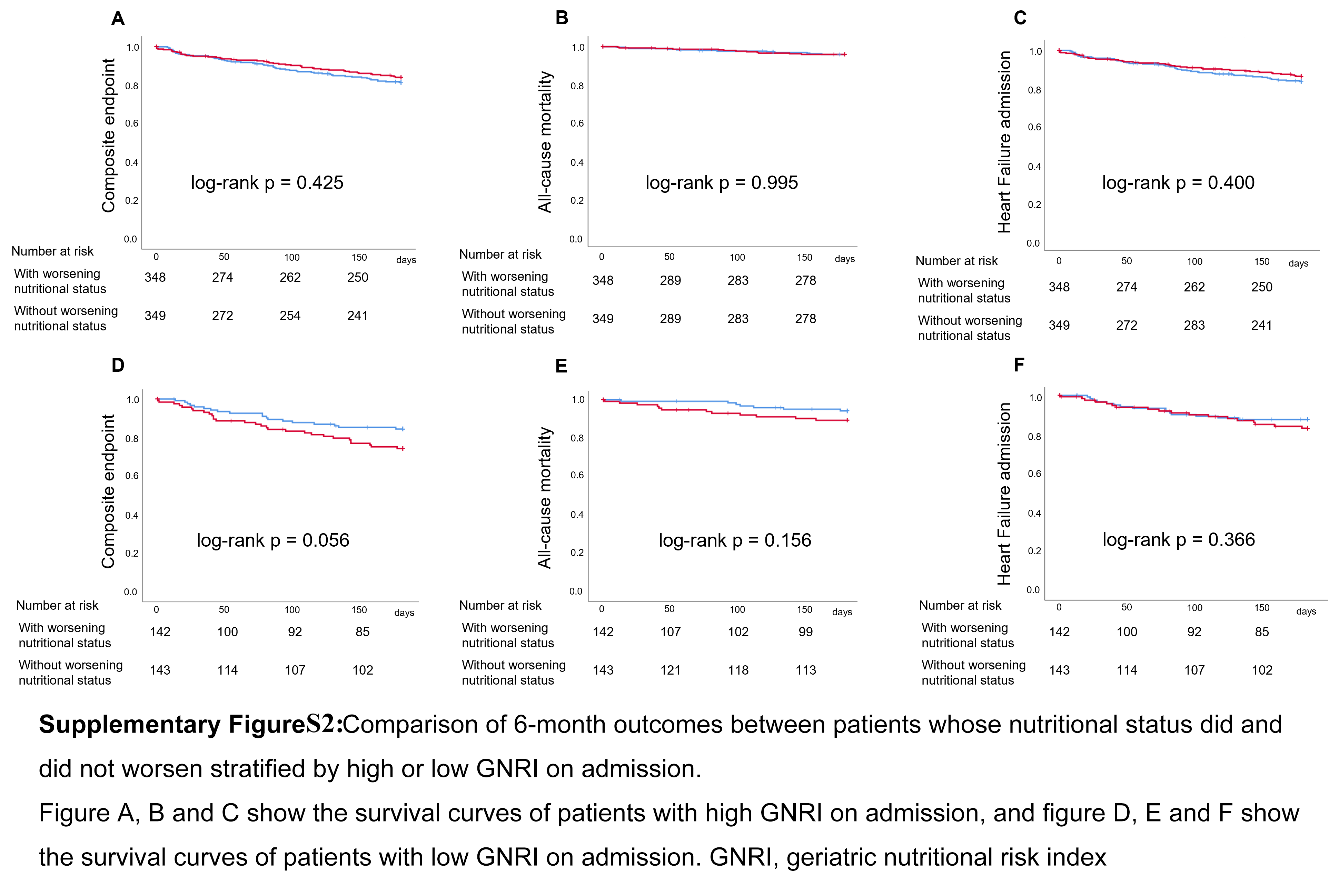

Supplement: Supplementary file 1 [file nutrients-14-04345-s001.zip › Suppl Figure S2_20220826.tif]
